# Designing nutrition-based interventional trials for the future: addressing the known knowns

**DOI:** 10.1186/s13054-019-2345-5

**Published:** 2019-02-19

**Authors:** Danielle E. Bear, Zudin A. Puthucheary

**Affiliations:** 1 Department of Nutrition and Dietetics, Guy’s and St Thomas’ NHS Foundation Trust, London, UK; 2 Department of Critical Care, Guy’s and St Thomas’ NHS Foundation Trust, London, UK; 3 Lane Fox Clinical Respiratory Physiology Research Unit, Guy’s and St Thomas’ NHS Foundation Trust, London, UK; 4 Centre for Human and Applied Physiological Sciences, King’s College London, London, UK; 5 William Harvey Research Institute, Barts and The London School of Medicine & Dentistry, Queen Mary University of London, London, UK; 6 Adult Critical Care Unit, Royal London Hospital, Whitechapel, London, E1 1BB UK

**Keywords:** Randomised controlled trials, Nutrition, Trial design, Functional outcomes

## Abstract

The consistent decline in critical illness mortality has a significant effect on trial design, whereby either an improbable effect sizes or large number of patients are required.

The signal-to-noise ratio is of particular interest for the critically ill. When considering the potential *signal,* interventions need to match outcomes in regard to biological plausibility. Provision of nutrition is a complex decision with many underappreciated aspects of *noise*. However, a fundamental interaction is often not accounted for *time*.

Working as a community to evolve trial design will be our challenge for nutrition interventions in the critically ill for the future.

The last 10 years has seen a realisation that mortality may not be the most meaningful outcome measure for interventional trials in critically ill patients [1]. This is not to say that mortality is not important, or indeed fundamental, to our practice. However, the consistent decline in critical illness mortality has resulted in difficulty in demonstrating that an intervention is capable of reducing mortality. This has a significant effect on trial design, whereby to affect mortality, one needs either an improbable effect size or a large number of patients.

In 2001, Sackett described “the only formula” of physiological statistics (Fig. 1), whereby confidence (e.g. the narrowness of the confidence interval around the intervention effect expressed as absolute or relative risk reduction) is influenced by the signal-to-noise ratio (SNR) and the square root of the sample size [2]. The SNR aspect of the equation is of particular interest in randomised controlled trials (RCTs) of nutrition interventions in the critically ill as small changes in this would require large changes in sample size to maintain confidence.

**Fig. 1:**
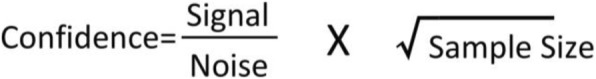
Sackett’s formula of physiological statistics

When considering the potential *signal*, it would seem appropriate to match the intervention to the outcome by strongly considering biological plausibility—that is, how likely it is that the intervention has the potential to influence the outcome to the expected degree [3]. In this regard, the biological plausibility that small alterations in protein/energy delivery or changes in the timing or mode of nutrition delivery will result in detectable changes in mortality is low. Our patients are subjected to many other potential threats to mortality, independent of nutrition. For this reason, discussions around more appropriate outcomes to measure include those related to muscle mass along with physical functional outcomes.

In their meta-analysis, Taverny et al. demonstrate that although both physical functional outcome measures and muscle mass are increasingly being used as primary outcomes, mortality still dominates as the choice for many prospective RCTs of nutrition in the critically ill [4]. Subsequently, they highlight the need for core-outcomes set for nutrition trials to be developed as in other specialist areas of critical care [5]. Aligning this with such initiatives already being developed for trials of physical rehabilitation [6] would seem sensible, allowing for a future of multiple interventions using both nutrition and physical rehabilitation.

As an intervention, provision of nutrition is frequently considered a simple therapy, but is in fact a complex decision between dose of nutrients, timing and route, all of which have to be decided upon based on the patient’s unique requirements. Significant amounts of important patient data are missing to make these decisions: accurate weight and height, body composition (e.g. fat mass vs. fat free mass), regular measurement of energy expenditure and prior/usual dietary intake. This final point is likely an important factor in determining response to nutrition interventions, but, similar to baseline physical function in trials of rehabilitation, is difficult to obtain. Fundamentally, nutritional trials in critical care examine what is delivered to the patient, not what they receive relative to their true requirement.

The above components are, in reality, increasingly understood, but underappreciated aspects of *noise* within nutrition RCTs. The greatest contributor to noise is collectively believed to be patient heterogeneity. In regard to nutrition trials, this would be in terms of baseline body composition, physical function and metabolism (which may be controlled for with larger (squared) sample sizes). More importantly, the response to nutrition interventions is likely to be heterogeneous too. Our lack of understanding of the complexities of metabolism and how these change over time in critically ill patients currently preclude appropriate trial design and sample size calculations.

Sackett’s model of physiology as applied to RCTs was not developed for trials in the critically ill. As such, a fundamental interaction is missing: *time*. This is perhaps where the equation fails as there is no modifier for how long an intervention might take to result in a detectable signal (Fig. 2)*.* Given the median length of stay for critically ill patients is around 1 week, most nutrition interventions in RCTs are only delivered for 6–7 days which is unlikely to result in measurable benefit. Using muscle mass as an example to illustrate this point, changes in muscle protein synthesis or breakdown may be seen immediately after an appropriate dose of amino acids, but for this to translate into changes in muscle mass that can be measured by other means (e.g. muscle ultrasound), a much longer duration is required [7, 8].

**Fig. 2:**
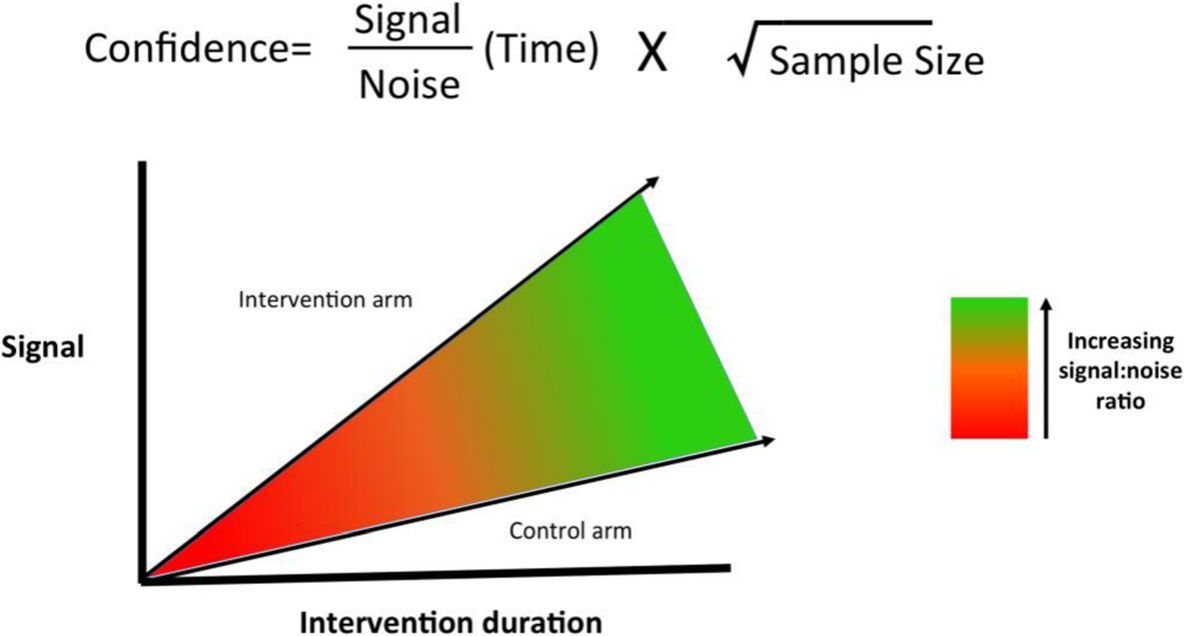
Signal/noise ratio as a function of time—Sackett’s modified equation

Timing and duration of the intervention in relation to the time point the outcome is measured is also important. All of the above points can be highlighted using recent, important, high-quality RCTs of nutrition interventions in critical illness. The TARGET trial demonstrated that providing 30.2 kcal/kg/day as opposed to 21.9 kcal/kg/day had no effect on any outcomes in any subgroup [9]. Randomising 4000 patients, a number that could be considered large enough to overcome all heterogeneity issues, may not have overcome the fact that it is implausible for a short intervention (median 6 days) to affect a longer term outcome (90-day mortality). Similarly, EAT-ICU, another well-executed RCT further highlights the importance of considering the duration of the intervention [10]. A 7-day (mean) nutritional intervention was unlikely to detect 6-month changes in the physical component score of the SF-36 quality of life survey without considering post-ICU nutrition. This last point is a pitfall of nutrition trials that is increasingly being recognised and addressed in currently recruiting RCTs [11, 12].

Post hoc trial design critique is, of course, far easier than performing the trials themselves. However, working as a community to evolve trial design, integrating lessons learnt from past trials and addressing the emerging needs of our patients, will be our challenge for RCTs of nutrition interventions in the critically ill for the future.

## Abbreviations

ICU: Intensive care unit
RCT: Randomised controlled trials
SF-36: Short Form 36
SNR: Signal-to-noise ratio
